# Cerebral Venous Sinus Thrombosis in Adults with Prothrombotic Conditions: A Systematic Review and a Case from Our Institution

**DOI:** 10.7759/cureus.7654

**Published:** 2020-04-12

**Authors:** Jack Komro, Dawood Findakly

**Affiliations:** 1 Internal Medicine, Kirksville College of Osteopathic Medicine, A. T. Still University, Kirksville, USA; 2 Internal Medicine, Creighton University Arizona Health Education Alliance/Valleywise Health Medical Center, Phoenix, USA

**Keywords:** antithrombin iii, protein c, protein s, papilledema, systematic review, hematology, cerebral venous sinus thrombosis (cvst)

## Abstract

Cerebral venous sinus thrombosis (CVST) is a rare condition characterized by elevated intracranial pressure due to impaired cerebral venous drainage, potentially leading to life-threatening consequences. We searched the PubMed electronic database for ‘cerebral venous sinus thrombosis’ and ‘prothrombotic’ cases reported in adults (19+ years) and conducted a systematic review for the published literature in the English language pooled with a case from our institution. Data were analyzed regarding patient demographics, risk factors, clinical features, treatment modalities, and outcomes when available.

Thirty cases of CVST were identified (29 case reports, of whom two were described in a case series, and the one case from our institution). The patients’ mean age was 39 years (range: 19 - 65). The male: female ratio was 1.14:1. The majority (73.3%) had at least one preexisting risk factor, with prescription drug use being the most common risk factor (33.3%) shared among all patients. Most patients (83.3%) presented with at least two symptoms. The most common presenting symptoms were headache (70%), gastrointestinal disturbance (50%), and seizures (40%). Focal deficits (36.7%), vision disturbances (30%), and altered consciousness (20%) were the remaining presenting complaints. Twelve cases (40%) commented on papilledema, with 10 (83.3%) having papilledema present. Anticoagulation abnormalities were examined in 26 cases (86.7%), out of which four cases (15.4%) had isolated protein S (PS) deficiency, three cases (11.5%) had isolated antithrombin III (ATIII) deficiency, and one case (3.8%) had isolated protein C (PC) deficiency. The most common initial imaging modality (22 cases, 73.3%), and most commonly used overall (23 cases, 76.7%), was computed tomography (CT). Magnetic resonance imaging (MRI) was the second most common imaging modality for initial use (five cases, 16.7%), diagnosis or confirmation of CVST (eight cases, 26.7%), and overall (21 cases, 70%). Heparin treatment was involved in the treatment of 18 cases (60%), and warfarin treatment was used in 10 cases (33.3%). Heparin-warfarin combination treatment was utilized in eight cases (26.7%). Most patients survived (28 cases, 93.3%), while the two remaining patients died secondary to brain death from the CVST (6.7%).

The findings from this study highlight the clinical characteristics of CVST. Therefore, this study aims to increase awareness of this rare entity. Physicians should maintain a high index of suspicion in order to diagnose patients presenting in the proper clinical context, given this case shares various forms of presentations with other common clinical conditions but requires long-term anticoagulation.

## Introduction and background

Cerebral venous sinus thrombosis (CVST) is a relatively uncommon, but potentially life-threatening condition, that has variable and non-specific forms of clinical presentations [1-2]. Anticoagulants, mainly heparin agents, are used as first-line therapy, with most patients attaining an excellent response [3]. This study's objective is to review the patient characteristics, risk factors, clinical features, treatment modalities, and outcomes of CVST, a rare and life-threatening condition in patients with prothrombotic states. 

## Review

Methods

Search Strategy

The present study protocol adheres to the preferred reporting items for systematic reviews and meta-analyses (PRISMA) guidelines for reporting systematic review protocols. The PubMed database was searched for adults (≥ 19 years old) and case reports in English using the terms ‘cerebral sinus venous thrombosis’ and ‘prothrombotic’ as keywords. Reference lists were also examined to identify relevant case reports. All full-text published cases were selected, and the authors independently assessed cases for inclusion.

Data Extraction and Analysis

**Figure 1:**
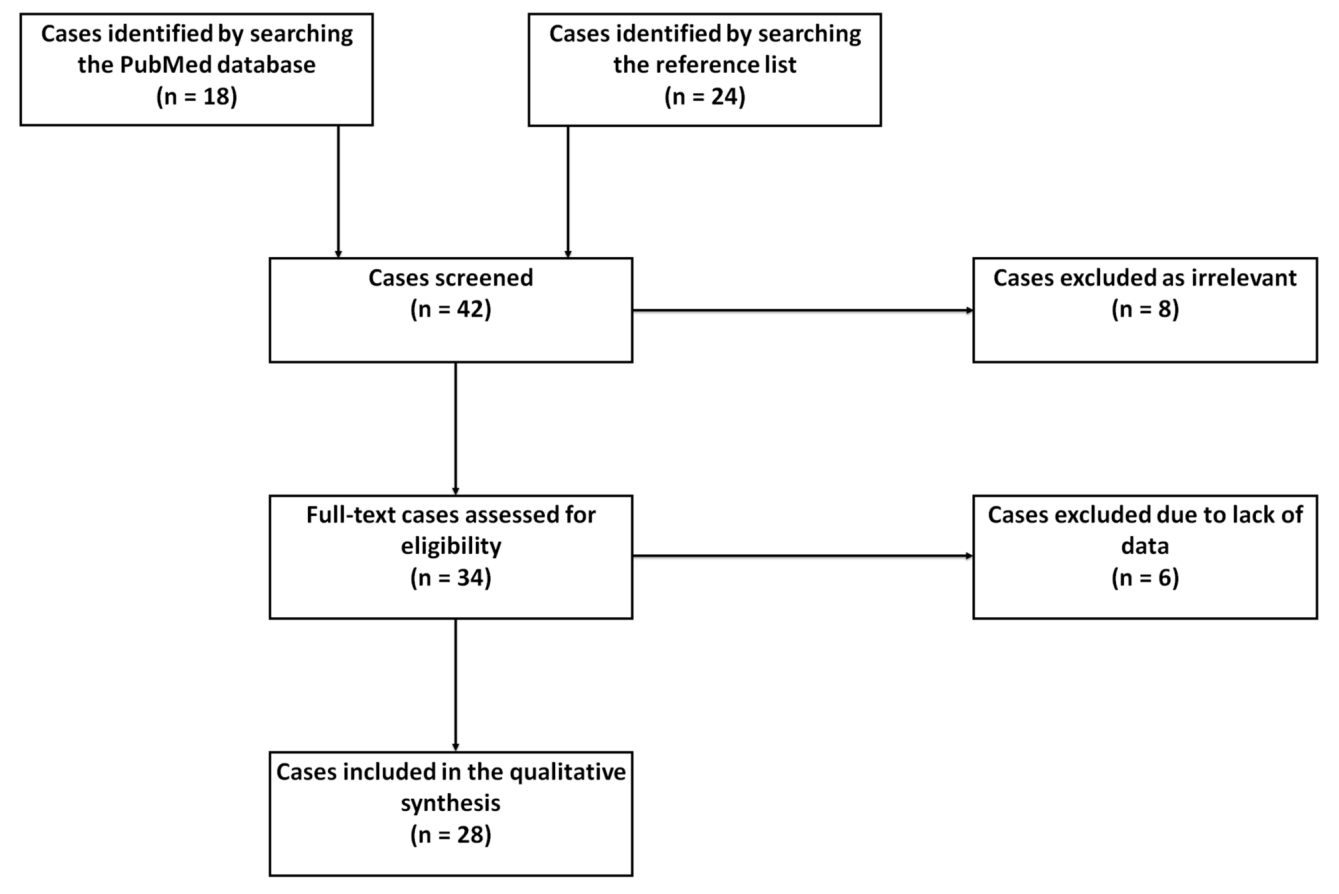
The PRISMA flow diagram for the systematic review detailing the association of CVST with prothrombotic abnormalities CVST: cerebral venous sinus thrombosis; PRISMA: preferred reporting items for systematic reviews and meta-analyses

All studies evaluating CVST with prothrombotic abnormalities were screened, with the selection of only those reports containing data on demographic information, clinical features, prothrombotic laboratory results, and diagnostic imaging. Unrelated case reports and those without prothrombotic lab results were excluded (Figure 1). Data are expressed in descriptive statistics using central tendency and dispersion measures.

Results

A total of 42 case reports of CVST with prothrombotic laboratory results were screened, with 28 publications ultimately included [4-31]. One case series described two cases, both of which were included, yielding 29 cases for this systematic review. With the addition of a case from our institution, a total of 30 case reports were analyzed. The demographics, clinical features, and outcomes of the 30 cases are summarized in Table 1.

**Table 1:** Summary of the Clinical Characteristics, Risk Factors, Diagnostic, Management, and Outcomes of CVST Case Reports Included in the Systematic Review AIHA: autoimmune hemolytic anemia; AMS: altered mental status; APLS: antiphospholipid syndrome; aPS: phosphatidylserine; aPS-Ab: antiphosphatidylserine antibodies; ARF: acute renal failure; AT: antithrombin; AT III: antithrombin III; BIW: two times weekly; b/l: bilateral; BRCA: breast cancer; CRP: C-reactive protein; cocci ag: coccidioides antigen; CT: computed tomography; CTA: computed tomography angiography; CVST: cerebral sinus venous thrombosis; DM: diabetes; d/t: due to; DTR: deep tendon reflex; DVT: deep venous thrombosis; EtOH: alcohol; F: female; fr: fracture; GBE: generalized body edema; GCS: Glasgow Coma Scale; GE: gastroenteritis; GTCS: generalized tonic-clonic seizure; HA: headache; HBV: hepatitis B virus; HCV: hepatitis C virus; Hcy: homocysteine; HHcy: hyperhomocysteinemia; HIT: heparin-induced thrombocytopenia; HIV: human immunodeficiency virus; HLD: hyperlipidemia; H/PF4-Ab: human platelet factor 4 antibody; HTN: hypertension; ICH: intracranial hemorrhage; ICP: intracranial pressure; ID: intellectual disability; IDA: iron deficiency anemia; IgG: immunoglobulin G; IgM: immunoglobulin M; ITP: immune thrombocytopenia; IVIG: intravenous immunoglobulin G; L: lumbar; LAC: lupus anticoagulant; LBP: low back pain; LDH: lactate dehydrogenase; LE: lower extremity; LMWH: low-molecular weight heparin; LOC: loss of consciousness; LP: lumbar puncture; LUE: left upper extremity; M: male; Mo: month; MRV: magnetic resonance venogram; MTHFR: methylene-tetrahydrofolate reductase; N: nausea; NR: not reported; NS: nephrotic syndrome; OCP: oral contraceptive use; PC: protein C; PE: pulmonary embolism; PS: protein S; R: right; RAEB: refractory anemia with excess of blasts; STEMI: ST-segment elevation myocardial infarction; TAT: thrombin-antithrombin III complex; T2DM: type 2 diabetes mellitus; TID: three times daily; UC: ulcerative colitis; UE: upper extremity; UFH: unfractionated heparin; US: ultrasound; V: vomiting; VUR: vesicoureteral reflux; w/o: without; Yr: year

Author (Yr)	Age (Yr) / Gender	Medical History	Duration of Symptoms Before Presentation	Risk Factors at Presentation	Presenting Symptoms	Initial Physical Examination Findings	Papilledema Present?	Anticoagulation Abnormality	Diagnostic Imaging	Treatments Provided	Follow-Up Period	Outcome	Complications	Final Diagnosis	Reference
Heistinger [4] (1992)	38 / F	Multiple episodes of UE phlebitis and DVTs post-delivery, appendectomy, tobacco	NR	OCP use x16 yr (interruptions during pregnancy), tobacco	GTCS; frontal cephalgia, N, V	Somnolent, meningism, anisocoria, reduced R corneal reflex, facial palsy, R arm brisk DTR	NR	Normal AT III, normal PC, decreased PS	Initial imaging: CT; Additional imaging: b/l carotid angiography (confirmed)	Heparin Phenprocoumon	NR	Alive	None	CVST d/t hereditary PS deficiency	4
Musio [5] (1993)	24 / M	UC	NR	UC x 24 mo	Hematochezia, weight loss, b/l frontal HAs, GTCS	Unable to complete full sentences or follow commands, R homonymous lower quadrantanopia, b/l dysmetria, gait disturbance, R LE weak, R foot clonus, R LE Babinski's reflex	Yes	Normal AT III, elevated PC, normal PS	Initial imaging: CT; Additional imaging: MRI (confirmed) SPECT scanning	IV methylprednisolone & oral sulfasalazine (for acute UC); phenytoin, anti-platelet therapy ,(aspirin) acetazolamide; recanalization of thrombosed venous sinus	NR	Alive	Hematochezia 2/2 acute UC, anemia requiring blood transfusion	CVST associated with UC	5
Tuite [6] (1993)	24 / M	Chronic LBP	NR	None	R arm focal motor seizures; HAs, neck pain, photophobia, N, V	Moderate expressive dysphasia, R facial palsy, R hemiparesis, unilateral sensory loss	NR	Decreased AT III, normal PC, normal PS	Initial imaging: CT brain; Additional imaging: b/l carotid angiogram (confirmed)	IV mannitol and dexamethasone, IV furosemide	NR	Brain dead, life support terminated	Elevated ICP, ARF	CVST d/t hereditary AT III deficiency	6
Vayá [7] (1995)	42 / M	Anemia	6 days	Anemia	AMS, pulsatile holocranial HA	No neurological deficits	Yes	Normal AT III, normal PC, normal PS	Initial imaging: CT; additional imaging: MRI (confirmed)	LP, corticoids, & mannitol (anti-edema), heparin	NR	Encephalic death	On 5th day of hospitalization patient developed mixed aphasia, b/l papilledema, supranuclear R facial palsy, and R UE hemiparesis b/l decerebration and Coma	CVST d/t RAEB	7
Akatsu [8] (1997)	65 / F	Appendectomy, lipoma removal, former tobacco	6 days	Daly PO estrogen, monthly IM medroxyprogesterone, former tobacco	GBE, R-sided HA, N, b/l arm w/t/n	b/l LE and UE pitting edema, L hand weak	No	Decreased AT III, normal PC, normal PS	Initial imaging: CXR; additional imaging: CT, MRI & contrast angiogram (confirmed)	AT III supplementation, warfarin, LMWH, cyclosporin	NR	Alive	None	CVST d/t NS	8
Lefebvre [9] (1998)	32 / F	Chonic HAs, mild ID	7 days	OCP x1 mo	N, hypersomnia, bifrontal HA	AMS, global hypotonia, b/l LE, reduced DTR, R LE Babinski's sign	Fundoscopy showed venous dilatation and blurring of nasal edge of optic disc. No specific report on papilledema	Normal AT III, decreased PC, decreased PS. Positive LAC, increased CRP, D-dimer, and LDH	Initial imaging: CT head (confirmed dx after it was re-examined after performing cerebral arteriography); additional imaging: cerebral arteriography MRI	Heparin oral anticoagulation	NR	Alive	Coma (prior to transfer), PE (10 days following admission), DVT R temporo-occipital hemorrhage	CVST d/t PC and PS deficiency	9
Singhal [10] (1999)	44 / M	Cirrhosis from HCV and EtOH use	NR	Cirrhosis	Generalized tonic-clonic seizure	Icteric	NR	Decreased AT III, decreased PC, decreased PS	Initial imaging: CT head; additional imaging: MRI with gadolinium CTV (confirmed)	Phenytoin Sodium LMWH Liver transplant	NR	Alive	None	CVST d/t AT III, PC, PS deficiencies d/t cirrhosis	10
Boulos [11] (1999)	42 / M	3000 meters high altitude hike one day prior	1 day	Recent high altitude hike	Several tonic-clonic seizures R facial droop R-sided hemiplegia Aphasia	R facial palsy, R hemiplegia, aphasia	Yes	Normal AT III, decreased PC, normal PS	Initial imaging: CT head Additional imaging: MRI (confirmed)	Heparin Valproate	NR	Alive	NR	CVST d/t isolated PC deficiency precipitated by high altitude	11
Akdal [12] (2001)	40 / F	BRCA post-radical mastectomy, tamoxifen	10 days	Tamoxifen	HA, L-sided weakness	L hemiparesis, positive Babinski sign	NR	Normal AT III, normal PC, normal PS	Initial imaging: CT; additional imaging: MRI (confirmed)	Anticoagulation	NR	Alive	NR	CVST d/t tamoxifen use	12
Rizzato [13] (2002)	24 / F	Puerperal	NR	Puerperal	Epileptic seizure; diffuse HA, R ear ache, N, V, mild L hemiparesis	NR	NR	Decreased AT III, MTHFR mutatation	Initial imaging: cerebral MRI, MRV	Anticoagulants, empiric antibiotics	NR	Alive	None	CVST d/t puerperium, AT III deficiency, and MTHFR mutation	13
Yilmaz [14] (2004)	19 / M	ITP, AIHA, splenectomy, chronic HBV, ICH	3 days	Evans syndrome (diagnosed at 5 years age)	Headahe, V, generalized convulsive seizure	Negative neurologic exam	NR	Normal AT III, normal PC, normal PS; normal factor VIII levels; positive prothrombin, G20210A heterozygous gene mutation	Initial imaging: CT; additional imaging: MRA (confirmed)	UFH, coumadin; anti-epileptic therapy; dexamethasone, acetazolamide	NR	Alive	NR	CVST d/t Evans Syndrome	14
Muthukumar [15] (2004)	38 / M	Motorcycle accident 24 days prior, brief LOC, two V episodes	NR	Recent closed head injury	Blurred vision Double vision on lateral gaze	Visual acuity 6/6, enlargement of blind spots on visual field exam, b/l lateral rectus (CN6) paresis	Yes	Normal AT III, decreased PC, decreased PS	Initial imaging: CT; additional imaging: MRA/MRV, digital subtraction angiography (confirmed)	Acetazolamide, phenindione	NR	Alive	NR	CVST d/t thrombophilia	15
Rufa [16] (2007)	57 / M	DVT, thrombosed hemorrhoids, episode of GE 1 hour prior to symptoms. Sildenafil use BIW for 1 year. Patient had prior episodes of thrombosis, occurred within 24 hours of Sildenafil use.	14 days	None	b/l blurred vision, occipital HA	Negative neurologic exam	Yes	Decreased AT III, normal PC, decreased PS. Increased fibrinogen, D-dimer	Initial imaging: carotid doppler; additional imaging: MRI brain (confirmed)	Sildenafil stopped, heparin, warfarin, acetazolamide	NR	Alive	NR	CVST d/t Sildenafil use	16
Ogata [17] (2008)	55 / M	IDA, melena	14 days	IDA	Melena, generalized seizures	L hemiparesis	NR	Normal antithrombin III, normal PC, normal PS, elevated D-dimer, TAT	Initial imaging: CT head; additional imaging: MRI (confirmed), cerebral angiography; upper GI endoscopy - open ulcers in stomach and duodenum; CT abdomen and chest, gallium scintigraphy MRV (4 weeks after dx - showed resolution of CVST)	Phenytoin Medical therapy for peptic ulcer, Iron supplementation	NR	Alive	None	CVST d/t IDA	17
Nayak [18] (2008)	36 / M	Single ectopic kidney with VUR leading to kidney transplantation 3 years prior. Immunosuppressive therapy - prednisolone, cyclosporine, azathioprine	2 days	Steroids	Continuous, diffuse HA worse in recumbent position and with coughing, blurred vision, V	Slightly irritable	Yes	Normal AT III, decreased PC, decreased PS	Initial imaging: MRV (confirmed)	Cephalosporin, dicoumarol	NR	Alive	NR	Idiopathic CVST after renal transplantation with preserved graft function	18
Sharpe [19] (2011)	25 / F	Inherited type 1 AT deficiency	4 days	3rd trimester pregnant, AT deficiency	HA, V	Negative neurologic exam	NR	Decreased AT III	Initial imaging: CT; additional imaging: MRI without gadolinium MRV (confirmed), Doppler US b/l LE (negative for DVT)	UFH antithrombin concentrate, C-section	NR	Alive	None	CVST d/t pregnancy, inherited type 1 AT deficiency	19
Nagesh Kumar [20] (2011)	35 / M	None	NR	None	Drowsy R-sided focal seizures; loss of consciousness	Unsteadiness	NR	Normal PC, decreased PS, normal Hcy	Initial imaging: CT brain; additional imaging: MRI brain, MRV (confirmed)	Phenytoin, LMWH, warfarin	NR	Alive	NR	CVST d/t PS deficiency	20
Skeik [21] (2012)	65 / M	Congenital hearing loss; obesity, former tobacco use	3 days	None	R-sided HA, flashing visual disturbance, imbalance L-sided neglect, disorientation, N, V	Extremities weakness, L facial palsy, LUE, and LE, brisk DTR, decreased L-sided sensation, L-sided body neglect, GCS 15	NR	Decreased AT III, decreased PC, normal PS	Initial imaging: CT head; additional imaging: MRI/MRA (confirmed), conventional angiogram, CTA	Primary venous mechanical thrombectomy (x2), heparin, aspirin (2/2 to possible HIT, so heparin stopped and started on aspirin)	NR	Alive	ICH, persistent mild amnesia	CVST complicated by ICH and mastoiditis with associated decreased AT III and PC	21
Kolacki [22] (2012)	28 / F	None	3 days	Vaginal NuvaRing (etonogestrel/ethinyl estradiol) started 18 days prior to onset of HA, previously intermittently used NuvaRing from 2002-2006 without complications	Severe, persistent b/l frontal HA Photophobia, N, V, posterior neck pain, stiffness	Stiff neck	NR	Normal AT III, normal PC, normal PS	Initial imaging: CT head; additional imaging: MRA (confirmed)	NuvaRing discontinued	18 days later - CT head showed resolution of hemorrhage and thrombosis	Alive	None	Acute hormone-induced CVST d/t NuvaRing use	22
Verma [23] (2012)	38 / M	None	14 days	None	HA, V, altered sensorium, L-sided body weakness	AMS, DTRs brisk	NR	Normal AT III, normal PC, decreased PS	Initial imaging: MRI, MRA; additional imaging: MRV (confirmed)	Enoxaparin, decongestive therapy; shifted from enoxaparin to warfarin	15 days later- repeat MRV showed recanalisation	Alive	None	CVST d/t PS deficiency	23
Sohoni [24] (2013)	36 / M	T2DM, migraine	4 days	Poorly controlled T2DM	Persistent L hemicranial HA	Unremarkable	No	Normal AT III, normal PC, normal PS	Initial imaging: MRI brain; additional imaging: MRV (confirmed)	UFH, LMWH	NR	Alive	None	CVST d/t uncontrolled T2DM	24
Costa [25] (2014)	30 / F	Chronic LBP, lumbar spine anatomic defects with transpedicular screw L4-L5-S1 fixation	NR	OCP, spinal surgery complicated by accidental durotomy	Severe HA, N, photophobia, R paresthesia	Wound dehiscence/infection, and CSF leakage, motor aphasia	Yes	Prothrombin G20210A heterozygosity, MTHFR homozygosity, HHcy	CT (confirmed)	Oral contraceptive pill stopped, warfarin	NR	Alive	NR	CVST d/t accidental durotomy during spinal surgery	25
Giraldo [26](2014)	47 / F	Tobacco	1 hour	Tobacco	HA behind R eye, unilateral extremity numbness	R hemiparesis, extremities motor deficits, R hemisensory loss to light touch	NR	Normal AT III, normal PC, normal PS, heterozygous prothrombin G20210A gene mutation	Initial imaging: CT head; additional imaging: MRI brain, gradient echo, MRA, MRV, catheter angiogram (diagnosed)	UFH, warfarin	6 weeks later - normal neurologic exam	Alive	NR	CVST d/t isolated thrombosis of L vein of Labbe associated with prothrombin G20210A gene mutation	26
Sugie [27] (2015)	41 / F	Migraine, obesity	8 weeks	None	b/l temporal HA, N, impaired vision, photophobia, extremity weakness	b/l abducens nerve palsy	Yes	Normal AT III, normal PC, elevated PS, elevated ESR, CRP	Initial imaging: MRI brain w/ gadolinium MRI aniography (MRA); additional imaging: MRV (confirmed), internal carotid angiogram	Glycerol, acetazolamide, LP	Heparin, warfarin, aspirin, rivaroxaban	Alive	NR	CVST-induced secondary intracranial hypertension secondary to APLS	27
Gleichgerrcht [28] (2017)	52 / F	HTN, HLD, fibromyalgia, EtOH, drug abuse	NR	Tobacco, trauma requiring orthopedic surgery 15-day exposure to prophylactic LMWH (leading to HIT and CVST)	Acute onset AMS on postoperative day 15 after T12 fr secondary to a motorcycle accident w/o head trauma	Negative neurologic exam	NR	Normal AT III, normal PC, normal PS Low platelets, positive H/PF4-Ab	Initial imaging: CT head, CTA; additional imaging: MRI brain, MRV (confirmed)	UFH, argatroban (switched from UFH after finding low platelets and positive H/PF4 antibody); dexamethasone (ITP treatment - failed); IVIG (ITP treatment - successfully normalized platelets)	None	Alive	HIT	HIT-induced CVST complicated by ITP	28
Qadir [29] (2017)	40 / F	Menometrorrhagia	3 days	Norethisterone TID for 21 days/month for the past 3 months	Jerky movements in L arm, HA	L UE decreased power	NR	Normal AT III, normal PC, normal PS, Factor 7 deficiency	Initial imaging: CT (confirmed)	Enoxaparin	NR	Alive	NR	CVST d/t Factor 7 deficiency possibly d/t OCP	29
Ganeshan [30] (2017)	21 / F	C-section (3 weeks prior), DVT (3 years prior)	< 24 hours	Puerperal	GTCS, HA, transient L arm numbness	Somnolent, pupils sluggishly reactive, L UE monoparesis with decreased DTR	NR	Elevated AT III, normal PC, decreased PS Elevated Hcy	Initial imaging: chest XR, echocardiogram, abdominal US; additional imaging: MRA (diagnosed)	Diazepam, phenytoin, methylprednisolone, ceftriaxone, acyclovir, phenobarbitone, mannitol, Fraxiparin, (LMWH) Levetiracetam	3 months later - repeat MRA showed restoration of normal blood flow through cerebral venous sinuses	Alive	Recurrent seizures and status epilepticus	CVST d/t puerperium, HHcy, recent C-section, suspected infection, low PS	30
Ganeshan [30] (2017)	25 / F	Migraine, C-section (3 years prior)	< 24 hours	OCP x2 yr, puerperal	Facial palsy, R arm weakness	Aphasic, R facial palsy, R UE weakness, decreased DTR	NR	Normal AT III, normal PC, normal PS, elevated Hcy	Initial imaging: CXR, echocardiogram, CT brain; additional imaging: MRI brain, MRV (confirmed)	Aspirin, oral contraceptive pills, stopped Fraxiparin, (LMWH), warfarin	4 months later - MRI brain and MRV showed resolution of CVST	Alive	NR	CVST d/t puerperium, HHcy, and OCP	30
Varner [31] (2018)	48 / M	Obesity	NR	None	GTCs, AMS	NR	NR	Normal AT III, normal PC, normal PS, positive IgM aPS	Initial imaging: CT, MRI, MRV, and conventional angiography (confirmed)	Levetiracetam, heparin, endovascular mechanical venous suction thrombectomy	3 months later presented for thrombophilia workup, pt was found to have a STEMI , was admitted and subsequently discharged after 5 days	Alive	None	CVST d/t aPS-Ab	31
Komro (2020) Present Study	61 / M	Essential HTN, pre-DM, obesity, negative cocci ag and IgG ab, negative HIV	7 days	None	Blurry vision	HTN	Yes	Decreased AT III, normal, PC, normal PS, negative prothrombin G20210A gene mutation, factor V Leiden mutation, antiphospholipid antibody	CT, CTV, MRI, MRV	Aspirin, acetazolamide, losartan	NR	Alive	None	Papilledema d/t increased ICP, possibly d/t CVST	Our case

**Figure 2:**
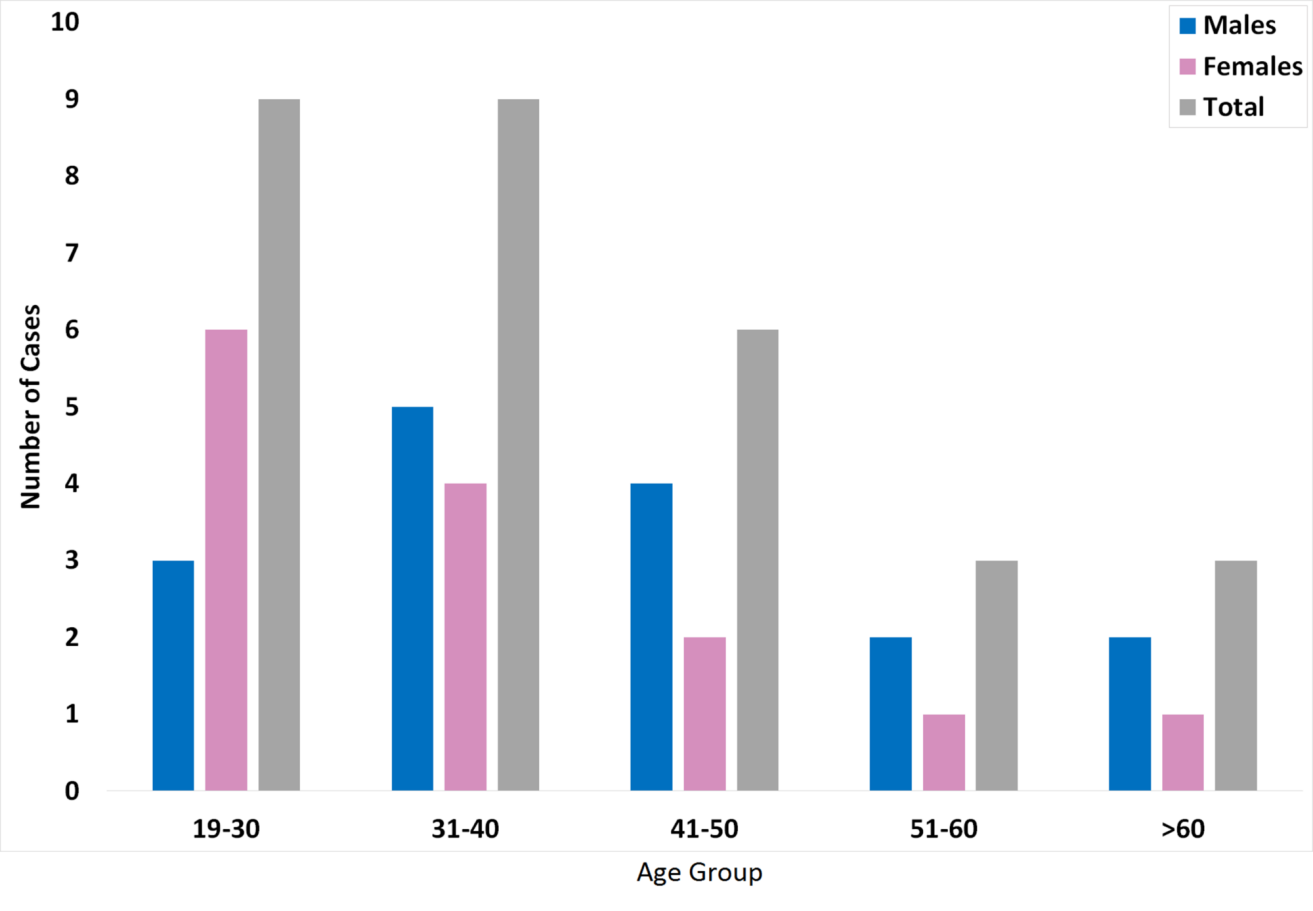
Age and gender distribution of adults with CVST. The bar graph represents the number of patients with CVST for the specific age and gender group

**Figure 3:**
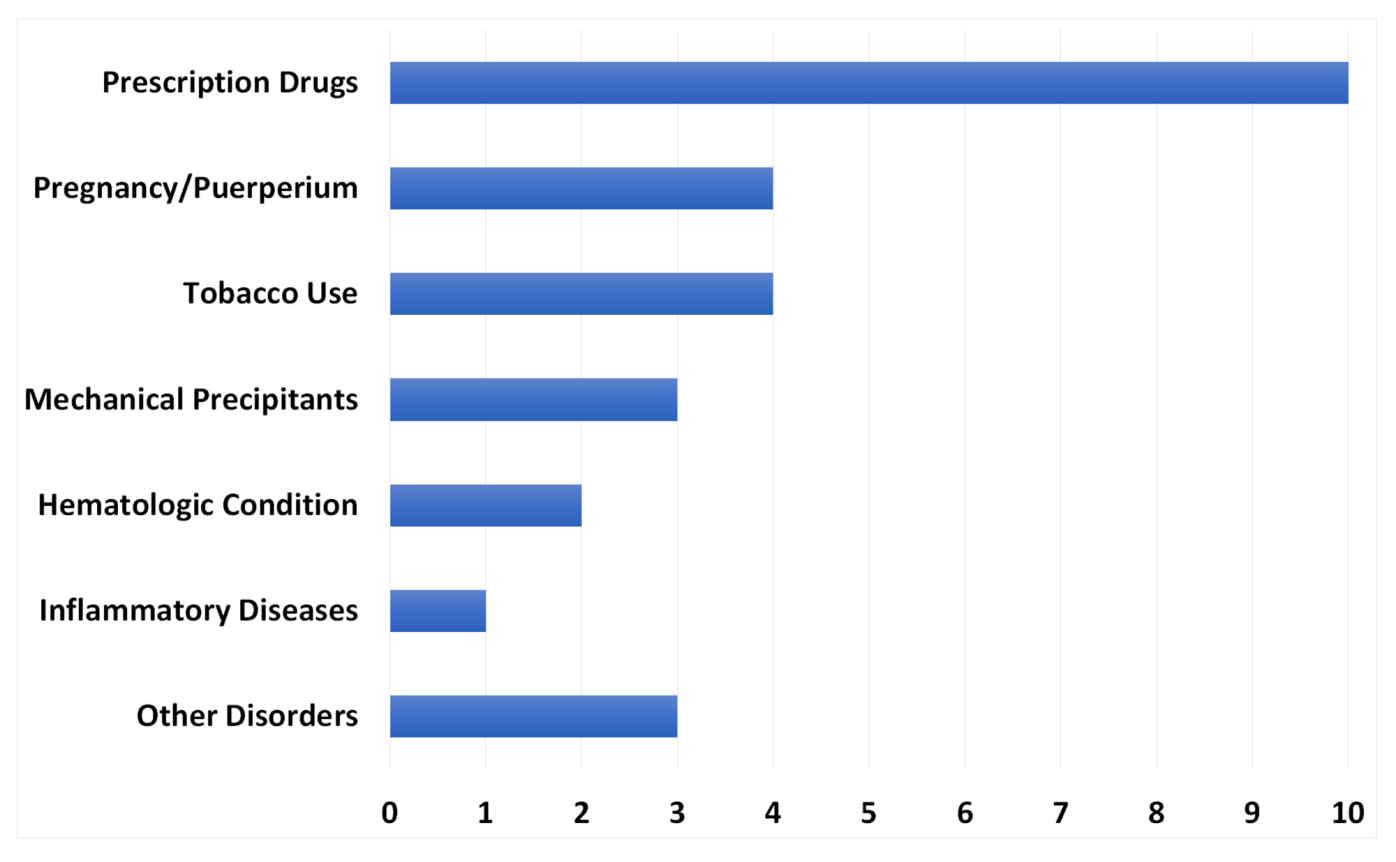
Risk factor frequency in 30 patients with cerebral sinus venous thrombosis (CVST)

The mean age at presentation was 39 years old (range: 19 - 65), with 24 (80%) being less than 50 years old. There were 16 male (53.3%) and 14 female (46.7%) patients (Figure 2). The majority (73.3%) had at least one preexisting risk factor (Figure 3). Prescription drugs were the most common risk factor (33.3%) shared among all patients. A history of tobacco smoking was reported in four cases (13.3%).

Among females, 11 (78.6%) reported having gender-specific risk factors. Six (54.5%) were receiving exogenous estrogen hormone therapy (EEHT), four (36.3%) were pregnant or puerperal patients, and one (9.1%) was receiving norethisterone therapy. Three case reports (10%) involved mechanical precipitants. Other disorders, including congenital heart disease, thyroid disease, Evans syndrome, diabetes, and cirrhosis, were reported in three cases (10%). The least common risk factors were a preexisting hematologic condition (two cases, 6.7%) or inflammatory disease (one case, 3.3%).

Of the 20 cases (66.7%) that reported the duration of symptoms, most (55%) had symptoms between two to seven days at presentation. Four patients (20%) presented earlier with symptoms up to one day, while five patients (25%) presented later with symptoms lasting at least eight days. One patient (5%) had symptoms more than two weeks, not presenting until two months after symptom onset (Table 2).

**Table 2:** The Duration of Symptoms Before the Patients’ Presentation

Duration	The fraction in each category (%)
0 - 1 day	4/20 (20.0%)
2 - 7 days	11/20 (55.0%)
8 - 14 days	4/20 (20.0%)
> 2 weeks	1/20 (5.0%)

**Figure 4:**
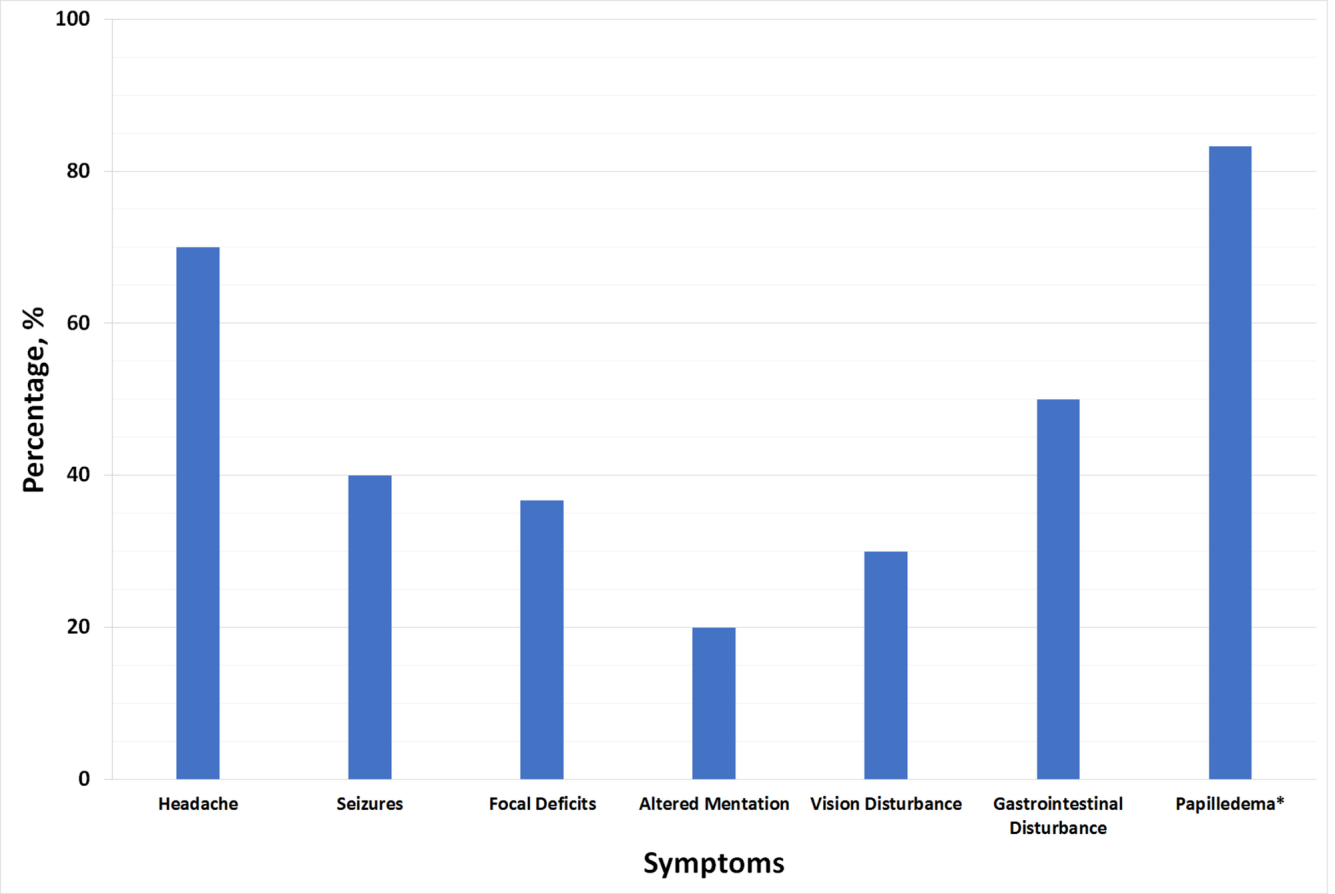
The presenting symptoms of cerebral venous sinus thrombosis (CVST) *The percentage of papilledema out of 12 cases with available data

Most patients (83.3%) presented with at least two symptoms (Figure 4). The most common presenting symptoms were headache (70%), gastrointestinal disturbance (50%), and seizures (40%). Focal deficits (36.7%), vision disturbances (30%), and altered consciousness / confusion / disorientation (20%) were the remaining presenting complaints. Twelve cases (40%) commented on papilledema, with 10 patients (83.3%) having papilledema present.

**Figure 5:**
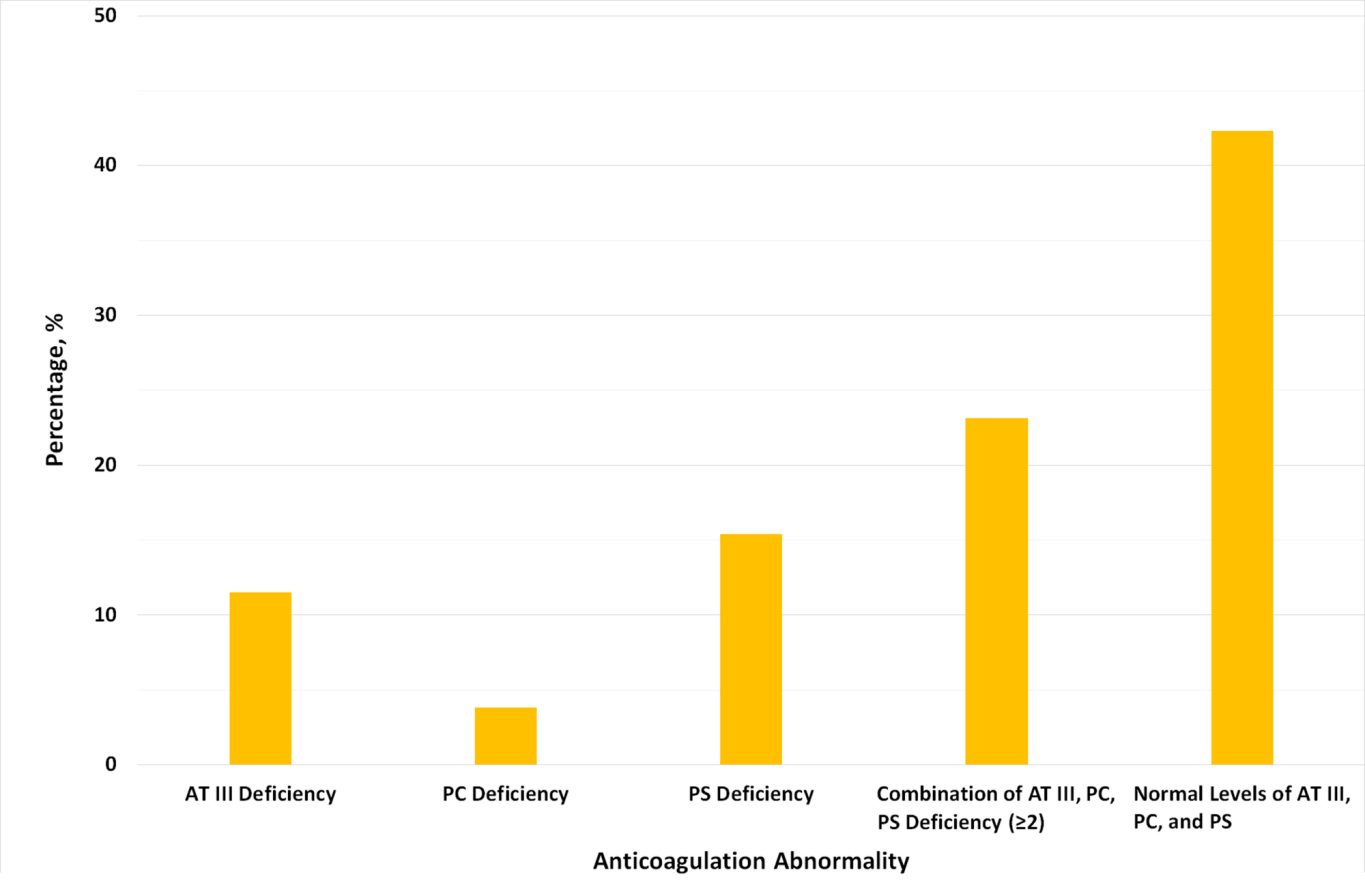
The prevalence of different prothrombotic conditions in patients with CVST AT III: antithrombin III; CVST: cerebral sinus venous thrombosis; PC: protein C; PS: protein S

Anticoagulation abnormalities were examined in 26 cases (86.7%). Four cases were excluded as they did not mention at least one of the following levels: antithrombin III (AT III), protein C (PC), or protein S (PS). AT III, PC, and PS were all normal in 11 cases (42.3%). An abnormality in at least two out of the three anticoagulants was reported in six patients (23.1%). Isolated AT III (three cases, 11.5%), PC (one case, 3.8%), or PS (four cases, 15.4%) deficiency was noted in the remaining cases (Figure 5).

Hyperhomocysteinemia (HHcy) was found among three (21.4%) female patients where it was associated with a G20210A prothrombin gene mutation in the first, low PS in second, and normal AT III, PC, and PS in the third patient. Moreover, among females with HHcy, one was puerperal, the second was using EEHT, and one was puerperal and had a two-year history of EEHT use.

The G20210A prothrombin gene mutation was found among three (9.4%) patients overall, out of which one was a male with low AT III and two were females (one with normal AT III, PC, and PS, and the other with no reported data on AT III, PC, and PS testing).

**Figure 6:**
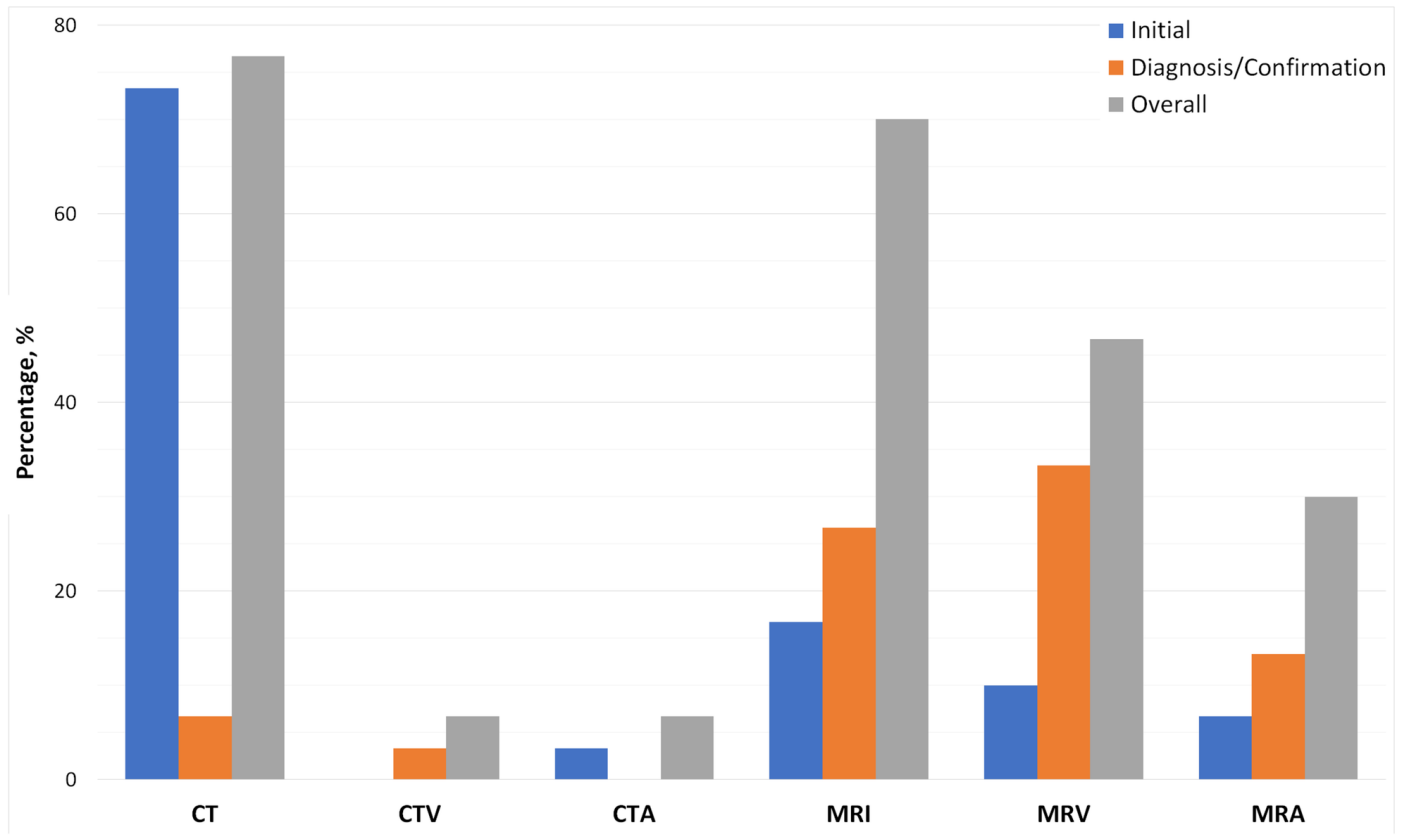
Imaging modalities used when evaluating patients with suspected CVST Initial (blue): first imaging used to evaluate the patients. Diagnosis/Confirmation (orange): the ultimately used imaging to diagnose or confirm the diagnosis of CVST. Overall (grey): the percentage of cases where imaging used at any point in patients’ evaluation CVST: cerebral sinus venous thrombosis

The most common initial imaging modality (22 cases, 73.3%) and most commonly used overall (23 cases, 76.7%) was computed tomography (CT) scan (Figure 6). Magnetic resonance venogram (MRV) was the most common modality that diagnosed or confirmed CVST (10 cases, 33.3%). Magnetic resonance imaging (MRI) was the second most common imaging modality for initial use (five cases, 16.7%), diagnosis or confirmation of CVST (eight cases, 26.7%), and overall (21 cases, 70%).

**Figure 7:**
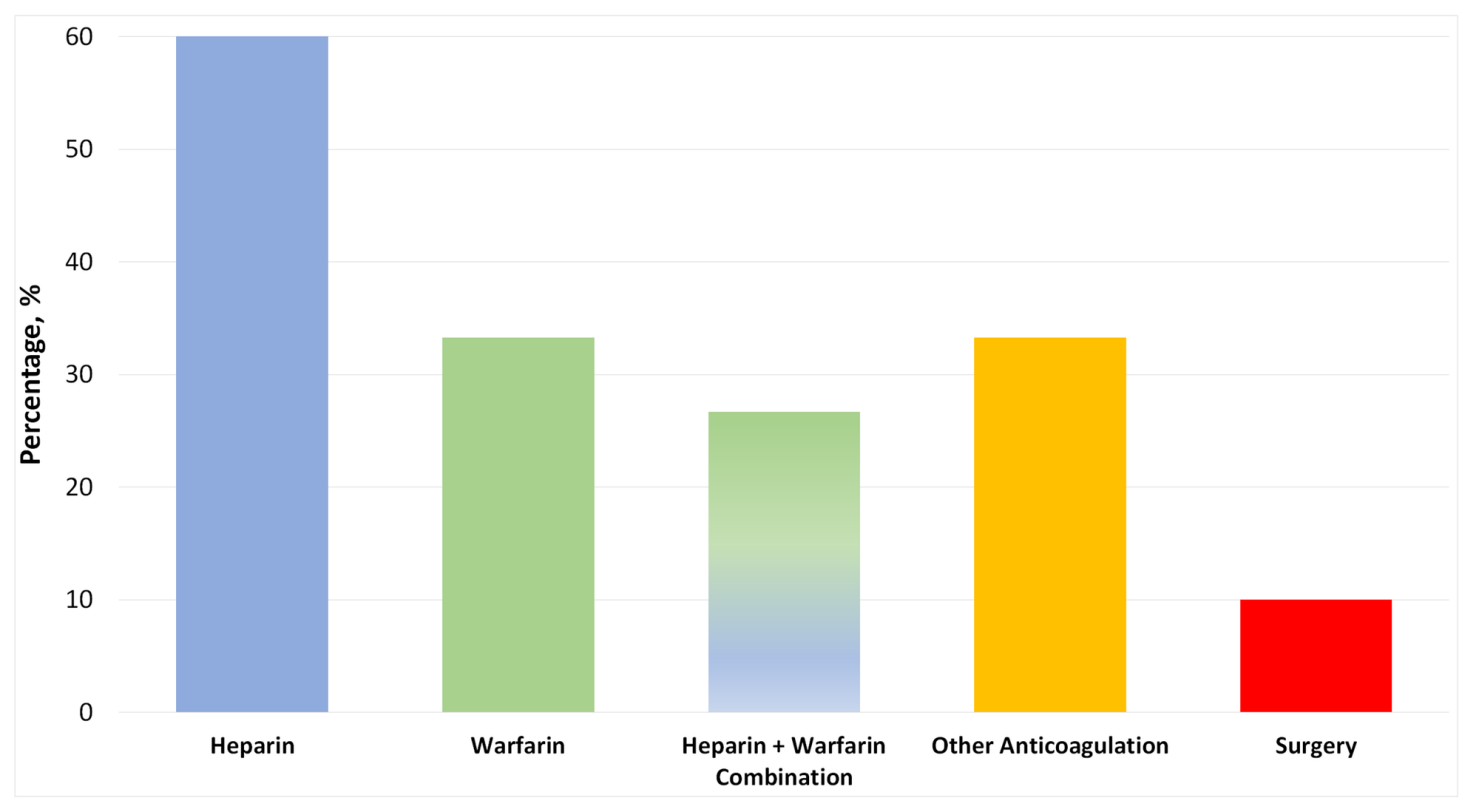
Treatments used for patients with cerebral sinus venous thrombosis (CVST)

Heparin agents were involved in the treatment of 18 cases (60%), and warfarin agents were used in 10 cases (33.3%). A heparin-warfarin combination treatment was utilized in eight cases (26.7%). Ten cases (33.3%) reported using other anticoagulants either with or without the use of heparin and/or warfarin agents (Figure 7). Surgical intervention occurred in three cases (10%).

Most patients survived (28 cases, 93.3%), while the two remaining patients died secondary to brain death from the CVST (6.7%). The two patients (100%) that died were administered mannitol and corticosteroids during their treatment course, and neither were given warfarin. One other patient who survived and recovered fully was given mannitol (Table 3).

**Table 3:** Survival outcome among cerebral sinus venous thrombosis (CVST) patients

Outcome	No. of patients/Total (%)
Alive	28/30 (93.3%)
Dead	2/30 (6.7%)

CVST occurs with similar frequency in males and females, and the symptom presentation often leads to a broad differential diagnosis.

Discussion

In this systematic review of CVST cases, several findings are notable. CVST is a rare condition that represents a unique challenge to physicians. It occurs at a similar frequency in both men and women and has a wide variety of symptoms that are clinically indistinguishable from other common clinical conditions, which most often lead to a broad differential diagnosis [1, 32-33].

Most cases had at least one preexisting risk factor indicating multifactorial etiology with multiple mechanisms involved in its pathogenesis. Prescription drug use was the most common risk factor, including those involved in oral contraceptive use. Among females, 10 (71.4%) reported having gender-specific risk factors. Four (40%) were pregnant or puerperal patients and six (60%) were getting EEHT.

Exogenous hormone therapy, pregnancy, and puerperium were the common risk factors for transient prothrombotic states and present in 78.6% of the female patients [34-36]. Tobacco use being the most common risk factor identified. More than half of the cases had symptoms between two to seven days before the presentation [37-38].

Most patients had symptoms for two to seven days at presentation and had at least two symptoms, with headache, gastrointestinal disturbance, and seizures being the most common presenting symptoms [39]. Normal AT III, PC, and PS were found in 42% of cases. Moreover, at least two of the three anticoagulants were deranged in a quarter of cases with available data [40].

The G20210A prothrombin gene mutation is linked with heightened risk for venous thrombosis, including CVST [40]. In this study, the G20210A prothrombin gene mutation was found in 9.4% of patients overall. Raised serum homocysteine levels are reported in the literature to cause a 4-fold higher risk of CVST [41]. In this study, HHcy was found among three (21.4%) female patients with a mean age of 25 years (range: 21 - 30).

CT scan was the initial modality of choice for most cases and the most commonly used overall, which could be due to its easy accessibility, relatively shorter scan period, and lower cost. MRI and MRV are the second and third most commonly used imaging overall, respectively. MRI and MRV were also the two most common imaging modalities used for diagnosis or confirmation of CVST. Therefore, MRV, in combination with MRI, is a non-invasive, specific modality that has proven reliable in diagnosing CVST [42-43].

Survival rate was 93.3%, and all deceased cases were not given warfarin during their treatment course. Papilledema (optic disc swelling due to high intracranial pressure) was present in 83.3% of the cases with available data. Therefore, looking for papilledema constitutes an essential factor which, unfortunately, was only reported in 40% of the cases.

## Conclusions

CVST may present with a variety of clinical presentations, which makes it a diagnostic dilemma and could lead to misdiagnosis or delayed diagnosis. Appropriate physical examination by primary care providers combined with a high index of suspicion, especially in the right context, is crucial in diagnosis. We advise for increased utilization of the direct ophthalmoscope to evaluate for papilledema in patients with suspected CVST. Further well-designed studies are warranted to help determine etiologies, as well as diagnostic and management strategies, for identifying CVST cases and to establish trends in patient outcomes.
